# Antimicrobial efficacy of corneal cross-linking in vitro and in vivo for Fusarium solani: a potential new treatment for fungal keratitis

**DOI:** 10.1186/s12886-018-0727-0

**Published:** 2018-03-02

**Authors:** Ziqian Zhu, Hongmin Zhang, Juan Yue, Susu Liu, Zhijie Li, Liya Wang

**Affiliations:** 1 People’s Hospital of Zhengzhou University and Henan Provincial People’s Hospital, Henan Eye Institute, Henan Eye Hospital, Zhengzhou, 450003 China; 2 Department of Pediatrics, Baylor College of Medicine, Houston, TX USA

**Keywords:** Antifungal therapeutic use, Corneal collagen cross-linking, Fungal keratitis, Fusarium solani, Mice

## Abstract

**Background:**

Fungal keratitis is one of the major causes of visual impairment worldwide. However, the effectiveness of corneal collagen cross-linking (CXL) for fungal keratitis remains controversial. In this study, we developed an in vitro and an in vivo models to assess the efficacy of CXL for Fusarium keratitis.

**Methods:**

The effect of in vitro CXL fungicidal was evaluated on the cultures of *Fusarium solani* which were exposed to irradiation for different durations. Viability of fungal was appraised under four conditions: no treatment (control); CXL: UVA (365 nm)/riboflavin; riboflavin and UVA (365 nm). Each batch of sterile plate culture was irradiated for different CXL durations.

The in vivo Therapeutic effect was studied on a mouse keratitis model. The animals were divided randomly into three groups: group A with no treatment (control); Group B with CXL treatment for two minutes and group C with CXL treatment for three minutes. The CXL procedure was performed 24 h post inoculation in each group. All mice with corneal involvement were scored daily for 7 days and 10 days after infection. Corneals were extracted at various time points for quantitative fungal recovery. Histological evaluations were conducted to calculate the number of polymorphonuclear cells.

**Results:**

Viability of fungal decreased significantly in CXL group with 30-min irradiation compared with that in control, riboflavin and UVA groups (*P < 0.01*).

The colony-forming units (CFUs) of fungal solutions in culture significantly decreased with CXL treatment (*P < 0.05*). Clinical scores, corneal lesion, corneal opacity, neovascularization and the depth of ulceration scores in group B and group C were remarkably lower than that in group A (*P < 0.05, P = 0.001, P = 0.001, P = 0.034* and *P* = 0.025 respectively). Scores of group C were much lower than that in group B. Histological revealed that destruction of corneal collagen fibers and infiltration of inflammatory cells into corneal tissue in group B and group C were much lower than that in group A.

**Conclusions:**

We believe that CXL treatment may be applied to fungal keratitis, therapeutic efficacy will improve with longer treatment duration.

## Background

Fungal keratitis(FK), a major eye disease, is one of the leading causes of severe visual loss. In all cases of infectious keratitis, fungal keratitis accounts for approximately 61.9% in North China [1], 39% in North India [2] and 6–20% in the United States [3]. Filamentous fungi (such as *fusarium solani*) are the key pathogens of fungal keratitis [4]. The overall prevalence of fungal keratitis has a clearly increasing trend in recent years. However, the therapy of fungal diseases of the eye is unsatisfactory due to several reasons: Therapeutic efficacy of traditional treatment such as eye drops is very limited in clinical because of poor bioavailability together with serious side effects [5]. It was also reported that 27% to 66% of patients with FK required surgical intervention [6]. Furthermore, eye banks cannot match demand, which is particularly common in most developing countries. Therefore, a new method of treatment is urgently needed to control the infection process more effectively.

Techniques such as Corneal Collagen Cross-Linking(CXL), a combination treatment with ultraviolet (UVA) light and riboflavin (vitamin B2) were proposed by Wollensak in 2003 [7], which has become an established treatment option for improving the biomechanical stability and resisting the progression of keratoconus. Further indications for the clinical use of CXL emerged rapidly since then, including Fuch’s corneal dystrophy [8], pseudophakic bullous keratopathy as well as infectious keratitis [9]. Recently the efficacy of CXL for the treatment was reported [10–12]**.** Although some cases of fungal keratitis were cured successfully, the efficacy of the CXL procedure in the management of fungal keratitis remains controversial [13–15].

In this study, we developed an in vitro and an in vivo model, to evaluate the inhibitory effects of the designed CXL against *F.solani* growth. We further explored the clinical and histological bio-pathology of mouse model of fungal keratitis to assess the anti-biofilm efficacy.

## Methods

### Fungi strains

*F.solani* (No.3.1791) was obtained from China General Microbiological Culture Collection Center (CGMCC, Beijing, China). After two or three subcultures, two milliliters of stroke-physiological saline solution(NS) were added to the slant medium containing well-grown *F. solani*. The fungal hyphae were ground with a sterile glass rod for making the fungal suspension, solution of which was adjusted by turbidimeter to get 0.5 Mx fungal suspension.

### In vitro viability test by CXL

Suspension of 10ul was placed to grow in each well of a sterile 96-well plate (Corning Life Science, Lowell, MA, USA) filled with 100 μl of potato dextrose agar (PDA, Beijing Sanyao Technique Development Co. China) per well for 24 h of incubation at 25 °C [16, 17]. The microplate wells have an internal diameter of 6.40 mm, so that the entire area can be exposed to UVA from 7.00 mm diameter light source.

Under sterile conditions, with the mass fraction of 0.1% riboflavin photosensitizer 20ul after 30-min, the cultures of *F.solani* were exposed to irradiation for different durations (respectively two, five, ten, 20 and 30 min). Further, the fungal viability in plate cultures irradiated with UVA (30 min) were evaluated under four conditions: no treatment (control), CXL: UVA (365 nm)/riboflavin, riboflavin and UVA (365 nm). Each process was repeated three times.

The standard procedure applied in the treatment of progressive keratoconus was followed to assess the antifungal effects of riboflavin and long-wave UVA irradiation. At a distance of 5 cm, fungal was exposed to laser irradiation. The size of the laser beam spot was 7 mm. The UVA (365 nm) has a power density of 45 mW/cm^2 and fungal beyond the irradiation scope was sheltered against light. After irradiation, the culture dishes were placed at 25 °C in an incubator for 48 h, photos of which were taken with a digital camera. Fungal gray values were analyzed by Image J software [18], and then the quantification of fungal cells viability was calculated.

### Animals

In total, 150male C57BL/6 J mice, weighing from 24 g to 27 g were used. The animals were fed and handled in strict compliance with the ARVO Statement For the Use of Animals in Ophthalmic and Vision Research. The study was approved by the Henan Eye Hospital Institutional Committee. All mice were anesthetized with an intraperitoneal injection of pentobarbital sodium (80 mg/kg.b.w.) (Sigmae Aldrich, USA) before interventions. Topical anesthetic (1% tetracaine hydrochloride drops) was applied for local anesthesia of corneal. The cornea of each mouse was scarified using a sterile scalpel to create a superficial wound of intersecting marks in a grid pattern, as referred [19]. A sharpened bamboo toothpick (0.30 mm tip diameter, 1.10 mm tip length) was used to scrape along the scratch 2 to 3 times to create a rough surface. The scarified cornea was subsequently smeared with fungi for fungal infection. A positive model was confirmed by an observation from a confocal microscope detection of fungal hyphae and/or spores 24 h post-inoculation.

### Corneal CXL treatment

The mice were divided randomly into three groups: group A. control group; group B. treated with CXL for two minutes; group C. treated with CXL for three minutes. Treatment started 24 h after fungal was smeared. The corneal epithelium of mice was scraped each group of 2-mm fields (which is a standard method). Then group B and group C were initiated CXL treatment. The lesions were instilled immediately with 0.1% riboflavin solution at 5-min intervals until a yellow dye in the aqueous humor were confirmed in the anterior chambers under a slit lamp with a cobalt blue filter. The corneas were continuously exposed to UVA irradiation (365 nm; irradiance 45 mW/cm^2; CCL-365VARIO instrument: guangteng technology xiamen co.LTD).

### Clinical examination

All mice were observed under a slit-lamp biomicroscopic and were scored accordance with the Schreiber scoring system [20] daily for 7 days and 10th day after modeling. Corneal lesion, the depth of ulceration, corneal opacity, edema, neovascularization were clinically evaluated on the 10 days. Corneal ulcers were graded as follows [21]: stage 0, no ulcer; stage 1, superficial ulcer; stage 2, medium-depth ulcer; stage 3, deep ulcer; stage 4, descemetocele; and stage 5, corneal perforation.

### Corneal fungal plate count

Ten mice were randomly sacrificed in each group in 1, 3, 7, 10 days after modeling respectively, eyes were enucleated for quantitative fungal recovery confirmed by plate counts [22], 0.85% of the stroke-physiological saline solution(NS) was added to the cornea which was grounded with a grinding stick to release the fungus. The suspension was centrifuged at 2000 rpm for 10 min for collecting supernatant, then cultured on potato dextrose agar(PDA) at 25 °C for 2 days, the quantity of colonies was determined in all cultures.

### Pathological observation of corneal tissue in mice

On the 10th day after treatment, mice went through cervical dislocation and the corneals were extracted for histopathological examination. The corneas of mice, with the mass fraction of 4% formaldehyde fixed after 24 h. Then the samples were embedded in paraffin and sagittally cut into 5-um-thick sections. Hematoxylin–eosin (HE) staining was used for histomorphological analysis of all groups. Observation was performed with the aid of a Nikon Eclipse E100 light microscope (Nikon, Sendai, Japan) under × 20 magnification. The disposition of collagen fibers, and inflammatory infiltration, were analyzed in terms of the microcorneal slices. Based on a scale of 0 to 4, the previously described inflammation score [23] was modified to the following: 0, no inflammation; 1, minimum change; 2. mild changes; 3. moderate changes; 4. severe changes.

### Statistical analyses

Statistical analyses were made by applying the triplicate values of each experimental condition. The data were expressed as the mean ± SD. Fungal gray values for different durations were compared with the control group was determined by T-test. Wilcoxon matched sign rank test for 2 related samples was used to compare the 30-min intra-group values. Clinical score and corneal fungal plate count obtained for each mouse were given One-way ANOVA for multiple comparisons. When homogeneity of variance was detected, the least significant difference (LSD) was used. In all other instances, Tamhane’s T2 test was performed. Pearson product correlation analysis was used to study the relationship between clinical score and corneal fungal plate count Nonparametric Kruskal–Wallis 1-way analysis of variance by rank were used for the corneal transformation. *P* < 0.05 was judged to be statistically significant. SPSS21.0 software (IBM, Inc., Chicago, IL, USA) was used for data analysis.

## Results

### In vitro

**Fig. 1:**
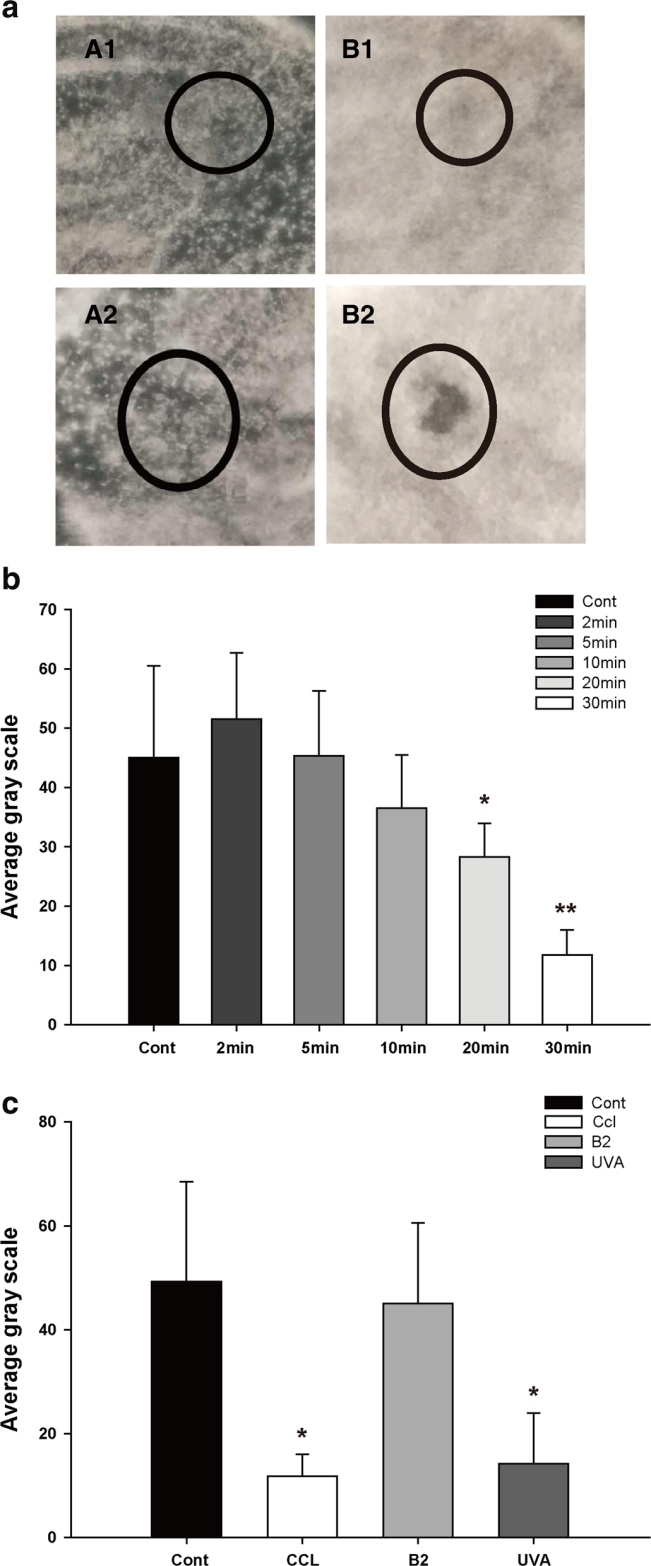
**a** The quantification of fungal cells viability obtained in culture treated with irradiation for 48 h. Black circles represent the radiation area, comparison before and after 10 min and 30 min CXL irradiation. **b** The fungal viability obtained was treated with different CXL time (2, 5, 10, 20 and 30 min). Statistically, there were big decreases in fungal viability with UVA (20, 30 min) compared to the control group, as assessed by the t-test. **c** The fungal viability irradiated with CXL (30 minutes) were evaluated under four conditions. Mean ± SD, *n* = 6, *p < 0.05 Vs Cont, **p < 0.01 Vs Cont

The fungal cells density was measured to determine the effect of CXL treatment on Fusarium. The strain under the CXL treatment exhibited a slightly lower growth rate than that without treatment (2 min: *P = 0.168;* 5 min: *P = 0.463;* 10 min: *P = 0.345;* 20 min: *P = 0.037;* 30 min: *P = 0.006*). Antifungal efficiency test of UVA on F. solani showed fungal cells density decreased significantly when the time of UVA irradiation increased (2、5、10、20 and 30 min) (Fig. 1a–c). The effect was maximal at 30 min (*P = 0.006*).

### In vivo

#### Clinical examination

**Fig. 2:**
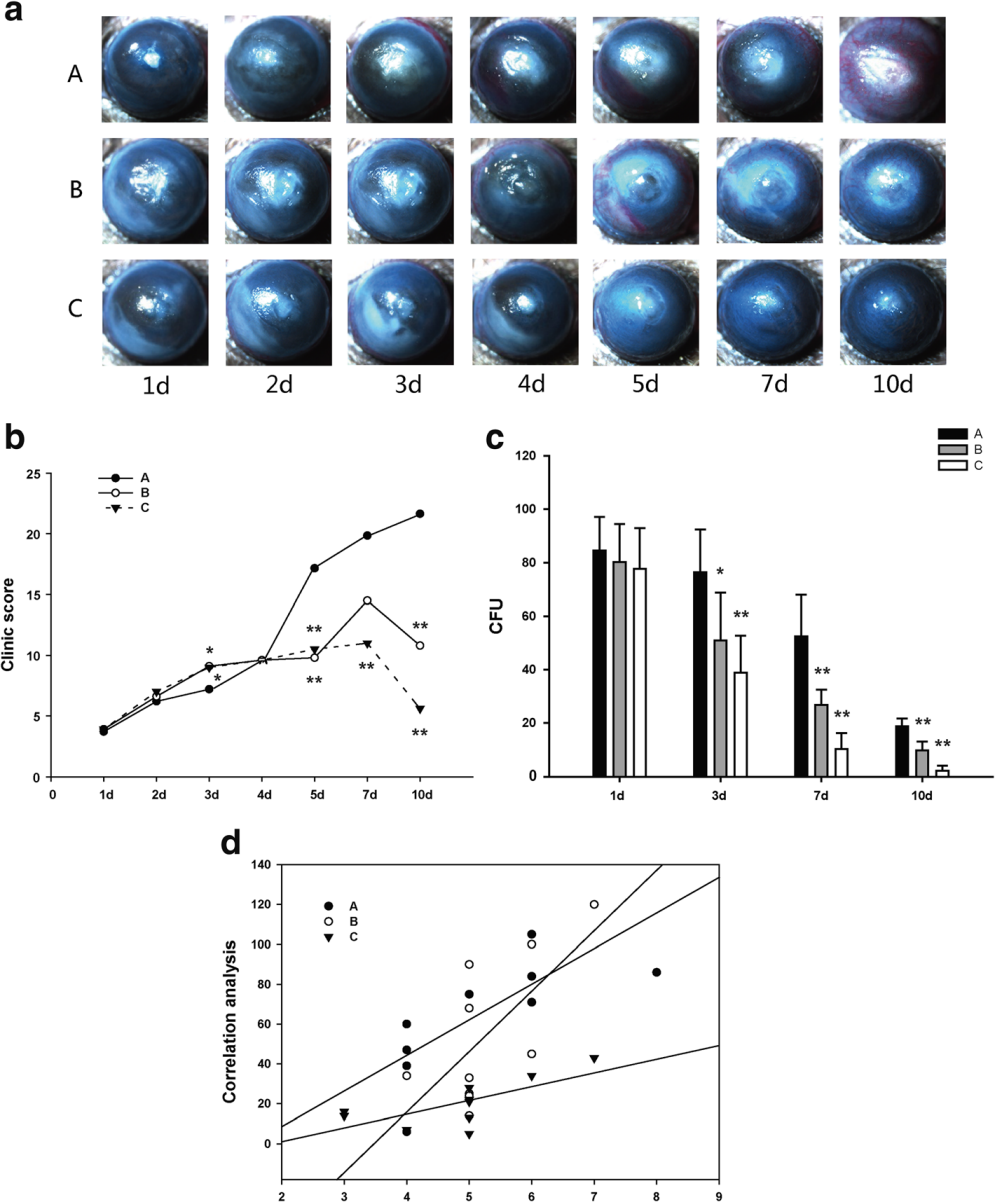
Eyes from each group after infection. **a** All mice were photographed every 24 h for seven successive days(× 16). **b** Efficacy of each treatment was evaluated under the Schreiber scoring system. There were severe corneal infiltrates, clouding and anterior chamber hypopyon in the group A. Statistically, there were big differences between Group B and Group C compared to Group A. Mean ± SD, *n* = 10, *p < 0.05 Vs Cont, **p < 0.01 Vs Cont, Group A: no treatment (control); Group B: CXL treatment for two minutes; Group C: CXL treatment for three minutes. **c** The CFUs was obtained in culture from each group, in 1 day, 3, 7,10 days after modeling. There was a statistically significant decrease in CFUs in both group B and group C compared to group A. *n* = 10, *p < 0.05 Vs Cont, **p < 0.01 Vs Cont, Group A: no treatment (control); Group B: CXL treatment for two minutes; Group C: CXL treatment for three minutes. **d** Scatter plots of correlation between corneal lesions and the fungal colonies in each group of mice. Mean ± SD, *n* = 10, *p < 0.05 Vs Cont, **p < 0.01 Vs Cont, Group A, control group; Group B, treated with CXL for 2 min; Group C, treated with CXL for 3 min

Clinical Schreiber scores obtained after infection indicated that there were statistically significant differences between CXL group and the control group (*P < 0.05*) (Fig. 2a, b). The longer the treatment time, the better keratitis recovered.

#### Corneal fungal plate count

There is a statistically significant difference between treatmen groups (*P* < 0.05) and Group A. The number of fungal colonies in Group C was much lower than that of the Group B**.** However, the difference was not statistically significant (Fig. 2c).

In addition, on the 3rd day after infection, pearson correlation analysis showed that the number of colonies in CXL group was positively correlated with the severity of corneal lesion group. A: *r = 0.771, P = 0.009;* group B: *r = 0.678, P = 0.031* whereas group C*: r = 0.707, P = 0.022,* see (Fig. 2d).

#### Histological examination

**Fig. 3:**
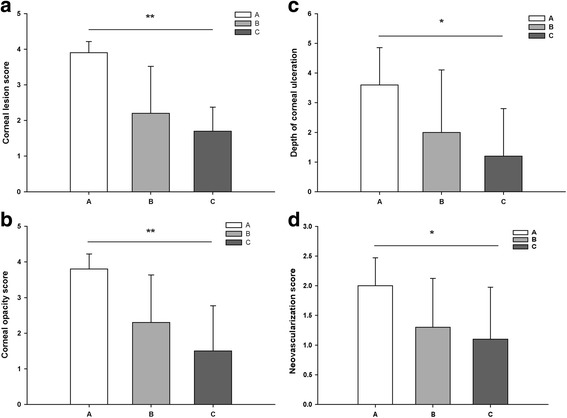
A comparison of the Clinical Findings on 14 day after treatment. **a** Corneal lesion (*P = 0.001*). **b** Corneal opacity (*P = 0.001*). **c** neovascularization (*P = 0.034*). **d** The depth of ulceration (*P = 0.025*), Mean ± SD, *n* = 10, *p < 0.05 Vs Cont, **p < 0.01 Vs Cont, Group A, control group; Group B, treated with CXL for 2 min; Group C, treated with CXL for 3 min

The clinical findings on the 10th day showed that the mean corneal lesion, corneal opacity, neovascularization and the depth of ulceration scores differed greatly among the control group(*P = 0.001, P = 0.001, P = 0.034* and *P = 0.025* respectively). In addition, group C apparently tended to have lower scores than group B (Fig. 3).

**Fig. 4:**
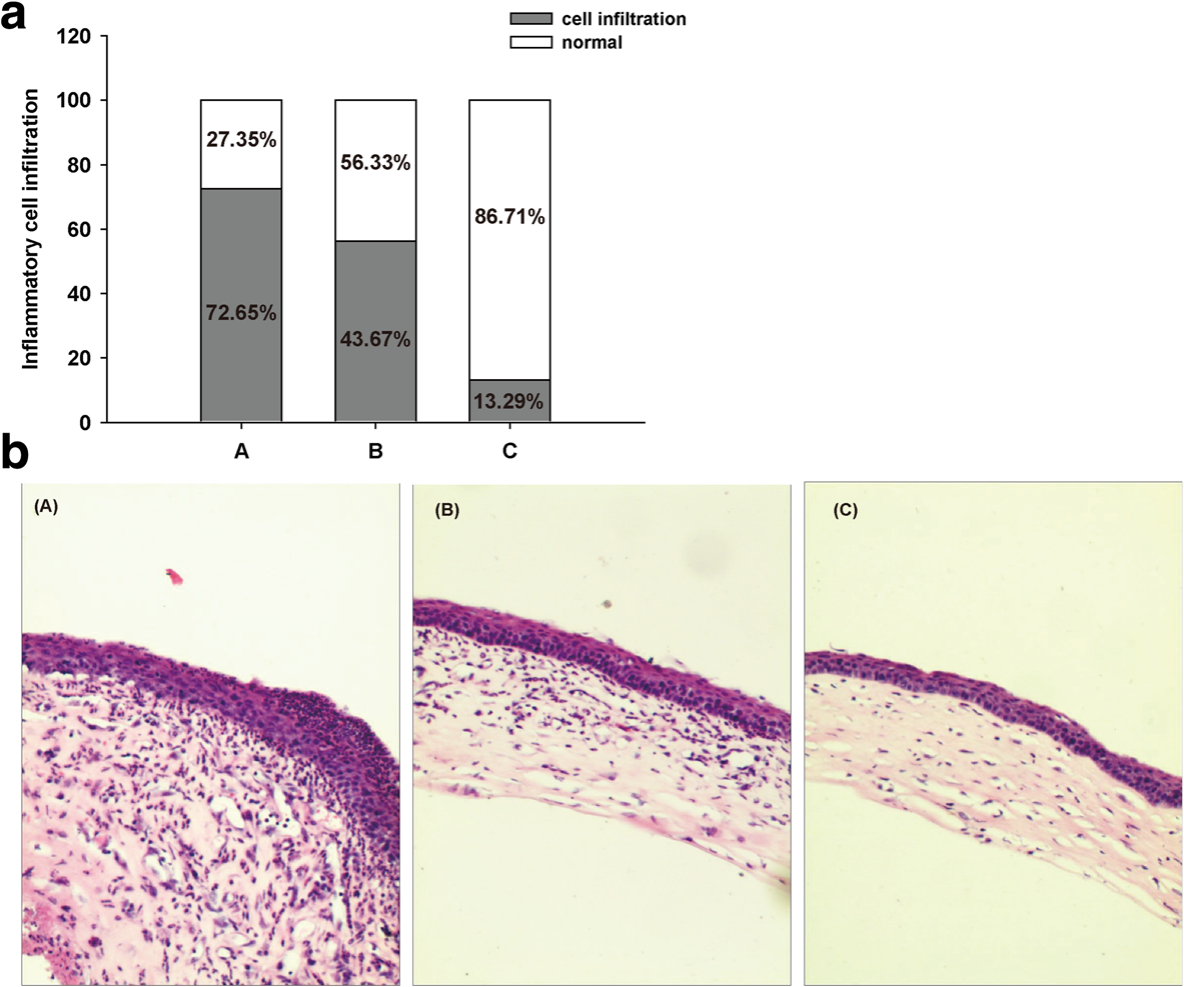
Histopathology of corneas in each group(× 20). **a** Comparison of inflammation cells infiltration among groups, (**b**). The corneal tissue of mice in each group was subject to HE staining. Group A, control group; Group B, treated with CXL for 2 min; Group C, treated with CXL for 3 min

In histological findings, there were severe inflammatory changes in Group A on the 10 days, inflammatory cells, and stromal edema were histologically evident within corneal tissue. Collagen destruction and sequence impairment were evident in the whole stroma, inflammatory cells accounted for *72.65%* of the total cells. In group B, collagen impairment was evident at the level of the anterior stroma. The inflammatory cells accounted for *43.67%* of the total cells. In group C, less severe inflammatory changes were noted on histopathological examination. The Inflammatory cells in corneal stroma were *13.29%*, as shown in (Fig. 4b).

## Discussion

Based upon the results of our in vitro study, we conclude that CXL could kill fungi effectively. Moreover, fungal activity was significantly reduced with a longer CXL irradiation time, which was consistent with the results of Jawaher [24]. However, the application of CXL in the therapeutic profile of fungal keratitis remains controversial. In 2013, shiwabuch [16] confirmed that CXL could inactivate Candida albicans in vitro fungal inactivation experiments, but there was no fungicidal effect against fusarium solani. It has been documented that fungal keratitis is caused by over 70 species covering 40 fungal genera [25], in this case, we believe that the sensitivity of the treatment differs, due to a wide variety of fungi species and the differences in their biological behaviors [26]. In addition, treatment sensitivity might also be related to the concentration of the solution. Demidova and Hamblin [27] also found that UVA only had an inactivation effect on the low concentration of Fungi. Furthermore, the treatment sensitivity seems relate to exposure-duration dependent pattern, as Makdoumi [28] revealed, with the energy density of 3 mW/cm^2, the 60 min exposure of bacterial suspension to UVA can totally inactivate the microorganism in solution, while 30 min exposure has limited effect of eradication. It is reported that fungal keratitis infection often exists as a biofilm, which is particularly difficult to clear because fungal cells are encapsulated in a protective and impermeable extracellular matrix (ECM) [4]. Thus, much higher doses of antimicrobials are required for biofilm clearance. According to the Bunsen-Roscoe rule [4], while maintaining the total energy, the absorption of light intensity is proportional to the time of irradiation and the effectiveness would increase with extension of irradiation duration. In conclusion, it could be hypothesized that the effect of CXL on inactivation of fungal pathogens is determined by the total energy provided by ultraviolet and riboflavin.

To obtain more evidence, the effect of CXL on fungal keratitis in vivo was further investigated. As the genes between mouse and humans are highly homologous, the mouse model is more conducive to assess the efficacy of CXL on fungal keratitis. Our findings suggested that with the increase of CXL time, the fungal colonies in treatment group decreased, the structure of corneal collagen fiber enhanced, the ability of corneal to resist edema increased, the digestion of corneal collagen suppressed, the progress of corneal ulcer delayed, the prognosis of mice better than that of the control group. Meanwhile, the therapeutical effect of CXL on fungal keratitis varied with different treatment time. Although the favorable outcomes obtained in this study are coincident with a certain of other laboratory studies and clinical studies [29], which report that CXL is effective in managing fungal keratitis, there are also studies obtaining contrary results to ours. Vajpayee [30] claimed that the practice of CXL combined with drug therapy did not increase the cure rate. After studying with 41 cases, he found that there was no significant difference between monotherapy on CXL and CXL combined with fungal regimen. Uddaraju [15] evaluated CXL curative effect on deep matrix of fungal keratitis, just to find that CXL group had a higher perforation rate than the control group. Most of the clinical researches aim at drug-resistant infectious keratitis for trial treatment. On account of the different time antimicrobial effects and different level of the keratitis, implement CXL for advanced progressive keratitis, further aggravated the severity of keratitis, late intervention may cut down the effectiveness of CXL treatment.

Basically, the effects of CXL inhibited fungal growth and infection may be related to the following factors: (1). Direct damage of Microorganisms by exposure to UVA radiation [31].(2). Riboflavin, as a photochemical medium, is non-toxic. Studies have shown that although the riboflavin could not inactivated fungi, it can induce the conformational changes of the component structure of fungal cell wall, which appears as the senescence of filamentous cells, decrease its cell size and increase its cytotoxicity [32, 33]. (3) Reactive oxygen species generate when riboflavin absorbs light and interacts with dissolved oxygen in solution damage the pathogen nucleic acids [34] (4). CXL reduce corneal melting and resist to enzymatic digestion, increase the strength and simultaneously reducing its penetrability by fungal hyphae [7, 35, 36]. Furthermore, after scraped corneal epithelium, CXL might entrap the fungal hyphae within the collagen matrix, thereby reducing the growth rate severity.

We found that CXL affected the various complications of fungal keratitis. Studies have suggested that CXL can activate fibroblasts, leading the increasing of ki-67 secretion, thereby increasing the degree of corneal opacity. However, the opacity of the CXL group was no different from that of the control group, which may be due to decreased α-SMA activation of fibroblasts [37]. The process of corneal healing may be associated with varying degrees of corneal neovascularization, thereby reducing the cornea transparency. Corneal neovascularization promotes the migration and recruitment of inflammatory cells into the lesion area, which is not only the key parts of the occurrence and maintenance of the entire inflammatory response, but also accompanying with pathological changes such as corneal neurodegeneration [38]. Chang and Bock [39, 40] believed that CXL can alleviate the pathologic neovascularization in the cornea of a mouse, but there is no research on fungal keratitis.

The results of our study showed that the number of corneal neovascularization and inflammatory cells decreased significantly after CXL. So we speculated that CXL increased the cornea toughness, reduced neovascularization invasion, thereby reducing inflammation response.

We should change the exposure time and wavelength of UVA, the concentration of riboflavin solution and the time of corneal action. Looking for the specificity of CXL for the treatment of different severity of keratitis with different pathological stages, in order to provide minimal side-effects on the healthy cells and tissue of the cornea.

## Conclusions

To sum up, the treatment with CXL is effective on inhibiting fusarium growth and causes symptomatic relief. But how can CXL coordinate the various processes and molecular mechanisms of the recovery of fungal keratitis, and further research is needed.

## Abbreviations

CXL: Corneal Collagen Cross-Linking
FK: Fungal keratitis
HE: Hematoxylin–eosin
NS: Stroke-physiological saline solution
PDA: Potato dextrose agar
UVA: Ultraviolet
